# Assessing the Feasibility and Acceptability of Virtual Reality for Remote Group-Mediated Physical Activity in Older Adults: Pilot Randomized Controlled Trial

**DOI:** 10.2196/53156

**Published:** 2024-11-08

**Authors:** Kyle Kershner, David Morton, Justin Robison, Kindia Williams N'dah, Jason Fanning

**Affiliations:** 1 College of Health Solutions Arizona State University Phoenix, AZ United States; 2 Department of Medicine Emory University School of Medicine Atlanta, GA United States; 3 Department of Health and Exercise Science Wake Forest University Winston-Salem, NC United States

**Keywords:** virtual reality, physical activity, videoconference, social connection, remote meeting, gerontology, physical inactivity, at-home intervention, descriptive statistics, eHealth, comorbidity, cybersickness

## Abstract

**Background:**

Physical inactivity represents a major health concern for older adults. Most social, at-home physical activity (PA) interventions use videoconference, email, or telephone communication for program delivery. However, evidence suggests that these platforms may hinder the social connection experienced by users. Recent advancements in virtual reality (VR) suggest that it may be a rich platform for social, at-home interventions because it offers legitimate options for intervention delivery and PA.

**Objective:**

This pilot study aims to determine the feasibility and acceptability of VR compared to videoconference as a medium for remote group-mediated behavioral intervention for older adults. The information generated from this investigation will inform the use of VR as a medium for intervention delivery.

**Methods:**

Nine low-active older adults (mean age 66.8, SD 4.8 y) were randomized to a 4-week home-based, group-mediated PA intervention delivered via VR or videoconference. Feasibility (ie, the total number of sessions attended and the number of VR accesses outside of scheduled meetings) and acceptability (ie, the number of participants reporting high levels of nausea, program evaluations using Likert-style prompts with responses ranging from –5=*very difficult or disconnected* to 5=*very easy or connected*, and participant feedback on immersion and social connection) are illustrated via descriptive statistics and quotes from open-ended responses.

**Results:**

None of the participants experienced *severe* VR-related sickness before randomization, with a low average sickness rating of 1.6 (SD 1.6) out of 27 points. Attendance rates for group meetings were 98% (59/60) and 96% (46/48) for the VR and videoconference groups, respectively. Outside of scheduled meeting times, participants reported a median of 5.5 (IQR 5.3-5.8, range 0-27) VR accesses throughout the entire intervention. Program evaluations suggested that participants felt personally connected to their peers (VR group: median 3.0, IQR 2.5-3.5; videoconference group: median 3.0, IQR 2.7-3.3), found that goals were easy to accomplish (VR group: median 3.0, IQR 2.8-3.3; videoconference group: median 3.0, IQR 2.6-3.4), and had ease in finding PA options (VR group: median 4.0, IQR 3.5-4.3; videoconference group: median 2.0, IQR 1.6-2.4) and engaging in meaningful dialogue with peers (VR group: median 4.0, IQR 4.0-4.0; videoconference group: median 3.5, IQR 3.3-3.8). Open-ended responses regarding VR use indicated increased immersion experiences and intrinsic motivation for PA.

**Conclusions:**

These findings suggest that VR may be a useful medium for social PA programming in older adults, given it was found to be feasible and acceptable in this sample. Importantly, all participants indicated low levels of VR-related sickness before randomization, and both groups demonstrated very high attendance at meetings with their groups and behavioral coaches, which is promising for using VR and videoconference in future interventions. Modifications for future iterations of similar interventions are provided. Further work using larger samples and longer follow-up durations is needed.

**Trial Registration:**

ClinicalTrials.gov NCT04756245; https://www.clinicaltrials.gov/study/NCT04756245

## Introduction

### Background

Physical inactivity is a global health issue. Recent studies identified that 7.2% of global all-cause mortality was due to physical inactivity and that 4 (coronary heart disease, type 2 diabetes, breast cancer, and colon cancer) out of the 5 leading causes of death could be combated by increasing overall physical activity (PA) [1,2]. Many of these disease states are associated with the aging process, and, unfortunately, older adults are the least active and second most obese segment of the population, exacerbating the impact of these conditions on quality of life and longevity [3,4]. Accumulating evidence suggests that older adults can improve quality of life and longevity by engaging in leisure-time moderate to vigorous PA (MVPA) as outlined in the national PA guidelines for Americans [5-7]. While the public widely knows the beneficial aspects of engaging in PA, multiple studies have found that less than half of Americans aged ≥65 years meet the national PA guidelines [8,9]. Global physical inactivity rose from 26.4% in 2010 to 31.3% in 2022, with no improvements in older adults’ MVPA behaviors [10]. This trend is likely due to multiple factors, significantly worsened by the COVID-19 pandemic, which further reduced PA levels in older adults [11,12]. Therefore, programs aimed at increasing MVPA in this group are urgently needed.

Theories integrating group dynamics as a tool for behavior change, such as social cognitive theory and self-determination theory (SDT), provide valuable frameworks for designing effective PA interventions across the lifespan [13-20]. However, a major limitation of many effective PA interventions is their reliance on in-person delivery, which restricts access for older adults who may face mobility challenges, transportation issues, and health concerns, preventing them from participating in programs that could benefit them the most. Our group has worked to blend in-person and videoconference delivery of group-mediated PA programs for older adults with chronic pain [21]. While this approach has been broadly effective, participant and interventionist interviews highlighted the “flattened” nature of the videoconference interactions, which limited participants’ sense of social connectivity. This sentiment has also been reflected in the literature on videoconference and social connection, highlighting the importance of one’s sense of physical proximity and “lifelike” interactions for developing new social bonds [22-26]. With more interventions targeting older adults’ MVPA moving to web-based designs accelerated by the COVID-19 pandemic, researchers must identify web-based formats that enhance the perceptions of physical proximity and, in turn, social connection [27,28].

Unfortunately, the key elements needed to create a sense of physical proximity or realistic interactions are inherently lost when using videoconference [26,29,30]. Researchers found that a gaze deviation (ie, attention diverted away from a person’s eyes or face) of 0.73° to 9.30° is noticeable to users and will affect their sense of social connection [25]. This gaze deviation is a given in videoconference meetings as users look away from the camera and toward other attendees. Some research suggests that videoconference-based communication may hinder the quality of interactions, with Tomprou et al [24] observing higher rates of interpersonal synchrony in facial and verbal expressions in audio-only communication compared to videoconference-based communication. The authors also found that videoconference meetings caused an increased rate of speaking-turn inequality (ie, 1 individual dominating the conversation), likely due to the loss of nonverbal cues such as eye contact, which are key for signaling turn taking [31]. With the increasing use of videoconferencing as a primary method for remote communication, Döring et al [29] investigated factors influencing the likelihood of experiencing videoconference fatigue. The authors found that videoconference fatigue is primarily driven by personal (eg, demographic, cognitive, or social), organizational (eg, number and duration of videoconference meetings), technological (eg, audiovisual fatigue), and environmental (eg, room setup and working from home vs office) factors. In summary, fostering remote social connections in interventions requires technologies that offer high-quality visual and audio cues, encourage rich discussions, and create a sense of shared physical space for participants.

**Figure 1:**
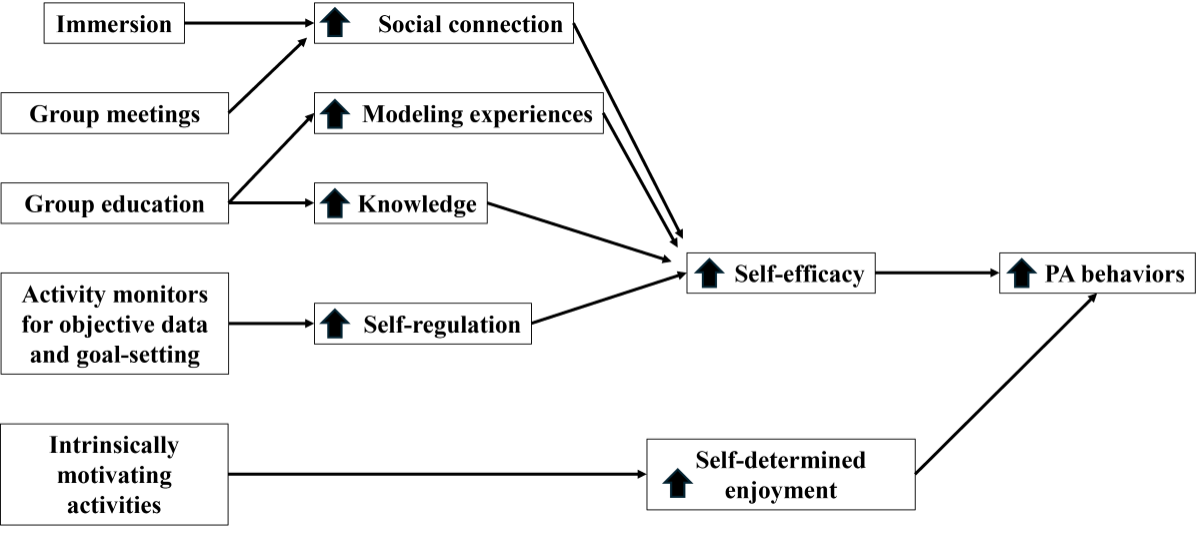
Virtual reality intervention model based on social cognitive theory and self-determination theory. PA: physical activity.

Fortunately, recent advances in virtual reality (VR), a form of extended reality (XR), suggest that it could be an effective medium for delivering immersive and scalable group-mediated interventions (Figure 1) [32-34]. Contemporary VR technologies cost approximately one-third of the manufacturer’s suggested retail price of new smartphones, and adoption rates for VR and other XR devices are increasing annually [35-38]. Popular headsets are wireless and do not require a high level of technological savviness. A key aspect of immersive VR design is maximizing the sense of place presence (ie, the feeling of being in a 3D space) [22,39]. In VR, users control actions with physical movements (eg, walking to retrieve a dropped ball and bending down to pick it up), which enhances the sense of place presence and immersion [40,41]. Contemporary VR technology also offers a large library of applications that require users to interact both socially and physically. Taken together, we expect that the availability of virtual meeting spaces and increased immersion in VR will better assist in developing social bonds within remote group-mediated interventions.

### Aim of This Paper

Given the novelty of VR as a health intervention platform, researchers should design interventions rapidly, iteratively, and carefully, sharing design choices and lessons learned to guide future researchers and developers [32,33]. The history of mobile health app development highlights the consequences of following commercial trends and traditional trial methods because most widely available behavior change apps lack a foundation in behavioral theories [42-45]. With VR shown to improve social connection and health outcomes in older adults, alongside its growing popularity, now is the time to establish effective methods for delivering socially rich PA interventions through this technology [46,47]. Therefore, the purpose of this paper is to describe the methods used to conduct the Virtually Engaging Socially with Physical Activity (VESPA) study*,* a pilot randomized controlled trial aimed at developing a VR-delivered, socially rich PA intervention for low-active older adults and comparing this program against a similar program delivered via videoconference. In addition to detailing the methods used, we will also present feasibility and acceptability results as well as baseline and follow-up measures on social connection, PA, and functional capacity; in addition, we will describe key modifications to be implemented in future VR-delivered PA interventions in low-active older adults.

## Methods

### Study Overview

The VESPA study was a pilot randomized controlled trial in which participants (n=9) engaged in a 4-week PA intervention delivered via either VR or videoconference. The trial was registered on ClinicalTrials.gov (NCT04756245). The results are presented according to the CONSORT-EHEALTH (Consolidated Standards of Reporting Trials of Electronic and Mobile Health Applications and Online Telehealth) checklist (Multimedia Appendix 1).

### Participants

Those who were eligible for the program were individuals aged ≥60 years who were classified as low active (ie, engaging in ≥30 min of moderate-intensity PA on ≤2 d/wk), had a BMI of 30 to 45 kg/m², were assessed as low fall risk by a personal physician, owned a smartphone with a wireless data plan, had access to at-home Wi-Fi, were willing to use a head-mounted VR headset, were willing to create or use a personal Meta account, were willing to wear a Garmin activity tracking watch, and were able to attend 3 in-person visits. Participants must have had no recent cardiac events or procedures, no uncontrolled hypertension or depression, no recent or ongoing treatment for cancer, and must have received physician approval before participation. Each participant was required to have a “designated VR space,” and an “activity buddy” (described in the VR Safety subsection). All participants were screened for cognitive impairment using the validated Modified Telephone Interview for Cognitive Status 21-point questionnaire, and those who received a score of <32 (out of 50) were excluded from the study [48]. In addition, given that previous experience with technologies may impact responses to VR in this study, participants were asked to report the average number of days they use various technologies (eg, laptop computer, tablet computer, and e-reader) and their self-efficacy for using these technologies on a scale ranging from 1 to 10 [49]. Finally, due to the extensive use of the VR headset, participants who indicated a *severe* rating on the Virtual Reality Sickness Questionnaire (VRSQ; discussed in the next subsection) were also excluded [50].

### Recruitment, Screening, and Randomization

Advertisements emphasized the opportunity for participants to meet with a behavioral coach and other group members via VR or other web-based media and engage in a 4-week PA program. Paper flyers were distributed to local businesses, clinics, and throughout Wake Forest University. All interested and potential participants were provided a full description of the study procedures and screened via telephone for eligibility. To ensure that participants were low active, the interested individuals were asked whether they engaged in moderate-intensity PA for ≥30 minutes on ≤2 days per week during this time. Those eligible were scheduled for an in-person visit to complete the informed consent document, a review of their health history, engage in the 6-minute walk test (6MWT), and provide written consent for the study team to contact their physician for their approval to participate [51]. After receipt of the approved physician consent, participants were scheduled for a second in-person visit where they completed self-report assessments, engaged in a “technology run-in” period, and learned their group allocation.

During the “technology run-in” period, participants engaged with the VR system (described in the Feasibility and Acceptability subsection under Methods). Immediately after the run-in process, each participant completed the VRSQ to assess VR-related sickness [50]. This was done to increase the data collected regarding VR-related acceptability. All participants who indicated a *severe* rating of VR-related sickness were documented and then excluded. By contrast, those who indicated any response other than *none* were asked whether the feeling of VR-related sickness would be too intense to continue with the program. After completing the VRSQ, eligible participants were randomized to their meeting medium (VR or videoconference) in a 1:1 ratio. The randomization sequence was generated using a random sequence generator, and study staff were blinded to the participants’ group allocations until the VRSQ was completed.

### Intervention

#### Key Intervention Tools

During the second visit, all eligible participants received an informational packet that included (1) information on the use of their meeting medium, (2) pre–group meeting reading and participation activities, and (3) an activity diary. The activity diary was 1 of the main components in which participants recorded their PA. The fields in the activity diary included the name of the activity, a description of the activity, the duration the participant spent engaging in the activity, the perceived enjoyment of the activity (rated on a scale ranging from –5=*did not enjoy* to 5=*enjoyed very much*), and the perceived exertion of the activity (rated on a scale ranging from 1=*no exertion* to 10=*high exertion*).

In addition to the informational packet, all eligible participants received a Garmin Vivosmart 4 activity tracking watch [52]. This activity monitor tracks PA via an embedded triaxial accelerometer paired with an optical heart rate sensor. Participants were instructed to wear the watch during all waking hours of the day for the duration of the program and to purposefully record each exercise bout on it (eg, by starting a “walk” activity). By doing so, the watch allowed the behavioral coach to closely examine the data before, during, and after each activity.

#### 4-Week PA Program

After a week of baseline PA data collection, participants engaged in a 4-week home-based, social PA intervention that was structured using aspects of social cognitive theory and SDT [13,14,21]. In both group-based and individual meetings with a behavioral coach, participants were encouraged to find intrinsically motivating activities that they believed would assist them in meeting the national aerobic activity guidelines [7,14]. During the group and individual behavioral coaching sessions, emphasis was placed on achieving a sufficient volume of PA while maximizing enjoyment. This was done to facilitate participant exploration of a variety of activity types, which they were encouraged to add to a “repertoire” of intrinsically motivating MVPA. Activities were determined as meeting the criteria for MVPA using heart rate data collected via the Garmin device. Heart rate ranges corresponding to moderate or vigorous intensities were determined using heart rate reserve calculated during the 6MWT (refer to the Measures section) [53].

Each group met using their assigned meeting medium for 45 to 60 minutes each week. Group meetings were designed to facilitate discussion regarding PA. To augment the meetings, an informational workbook was provided to the participants, and they were asked to read the content before each group meeting. The purpose of the workbook was to facilitate a discussion of the benefits of PA, barrier management, and the practice of skills required to successfully sustain behavior change. In concert with the workbook, the group itself was used as a tool for behavior change. Group members assisted their peers by providing insight into their own real-world and virtual PA, modifications, strategies for overcoming barriers, modeling, and verbal persuasion. The interventionist used the group discussion to promote enjoyable PA both virtually (VR or through videoconference) and in the real world. Each group member also individually met with an activity coach (ie, a graduate student studying health and exercise science with a bachelor’s degree in exercise science) in a tapered schedule, decreasing from 3 meetings during week 1 to 1 meeting during week 4. These meetings were designed to facilitate any technological troubleshooting and to engage in a discussion regarding participant PA. The activity coach reviewed the information received from the Garmin device and the activity diary, provided any necessary feedback on the exercise bouts, and offered suggestions for future PA. In total, each participant was scheduled to meet the activity coach individually 8 times and in a group 4 times.

#### VR System and Applications

The VR group used the Meta Quest 2, which is a wireless, head-mounted VR system that uses inside-out tracking (ie, cameras are embedded within the headset). These characteristics allow the user to interact with 3D objects and move freely within 3D spaces, constrained only by open real-world space of approximately 185 m^2^. Two handheld wireless controllers are used to interact with virtual environments [37]. To enhance the feeling of immersion, participants had the option to create an avatar in the meeting application (Spatial; Spatial Systems, Inc), which allows users to meet and interact in virtual conference rooms [54]. In addition, participants in the VR group were able to choose 3 to 4 applications from a list of PA applications requiring various intensities of exertion. These games included sports simulators, dance, and song-based activities, as well as other activities requiring large body movements [55-65]. Participants were encouraged to choose applications that they believed would be enjoyable and enhance their PA repertoire. Notably, VR was selected for use in this trial to enhance the perceptions of social connection. Thus, VR activities were offered as an opportunity to achieve PA, but participants were encouraged to develop an activity repertoire comprising any activities they preferred and to select activities based on daily preferences and barriers.

#### VR Safety

Multiple precautions were implemented to ensure participant safety during VR use. First, the Quest 2 has a built-in safety feature called the “guardian boundary” [37]. The guardian boundary is set up during the initialization process and whenever the Quest 2 is being used outside of the previous guardian boundary. The user can view their external environment from within the headset and scan their desired VR space for any impediments. A virtual boundary is then drawn, and approaching this boundary provides a visual cue in the headset to alert the individual to their position in space.

Participants randomized to the VR group were also required to have an “activity buddy” present during all VR-related PA. A study staff member met with the participants and their activity buddy via videoconference shortly after randomization to review the activity buddy’s role and discuss a proposed “designated VR space.” The purpose of the activity buddy was 2-fold: to provide verbal warnings and, in the event of an injury, to call for help. Activity buddies were instructed to verbally warn the participant if they approached the edge of their designated VR space and direct them back to the center of the area. In addition, they were provided with a protocol for evaluating the participant’s condition and, if necessary, calling for medical assistance. During the activity buddy meeting held via videoconference, a study staff member reviewed each participant’s proposed designated VR space, which was a clear 213 cm by 213 cm space in their home where they could engage in PA unobstructed. During this meeting, the study staff member either approved the participant’s designated VR space or provided recommendations on how to create a safer VR use environment. The study staff member ensured that the activity buddy knew the location of the designated VR space so that they could provide timely verbal warnings when the participant approached the guardian boundary.

#### Videoconference Group

The videoconference group met via a secure version of Zoom (Zoom Video Communications, Inc) [66]. This 2D application is widely used for videoconferencing and offers the ability for both small and large groups to meet as well as the formation of “breakout rooms” for private conversations during larger group meetings. All meetings were password protected and used the “waiting room” feature, which requires the meeting host to admit users, ensuring that only the videoconference participants were permitted in the meeting. Participants in the videoconference group were recommended to use PA videos available on the internet and real-world PA to accomplish their PA goals.

### Measures

#### Overview

All measures were taken during the first 2 eligibility visits and within a maximum of 2 weeks after the 4-week intervention. Baseline measures were collected by study staff members who were blinded to participant group allocation. However, the staff members conducting the follow-up assessments were aware of the participants’ group assignments. The measures of functional performance were obtained by trained study staff. To avoid social desirability bias, questionnaires that were more sensitive in nature, such as the Basic Psychological Need Satisfaction and Frustration Scale, were self-administered by the participants, with study staff present to answer any questions if needed [67]. All secondary outcomes were assessed at baseline and within a maximum of 1 week after the cessation of the intervention.

#### Feasibility and Acceptability

The feasibility of delivering a 4-week, at-home PA program was determined by the number of VR coaching sessions attended and the total number of self-reported VR accesses outside of coaching sessions throughout the program. The acceptability of delivering the PA program was assessed primarily via Likert-style questions ranging from –5=*very difficult or very disconnected* to 5=*very easy or very connected* and open-response feedback via pencil and paper and transcribed verbatim by a study staff member. These queries pertained to the technology used, social connection experienced, activity coaching, and ease in identifying intrinsically motivating activities. A second key domain of acceptability was VR-associated sickness, which is a common concern among those interested in using VR technology [40,68]. Thus, we assessed VR-associated sickness in all participants before randomization. This decision was made to maximize the data regarding the acceptability of VR use in this population. To do so, we used the 9-item VRSQ [50]. To be educated about, and properly exposed to, different VR spaces, all participants engaged in a tutorial application, the behavioral meeting application (Spatial), and a popular active game. This “technology run-in” period was designed to last approximately 30 minutes or until participants chose to discontinue. After this initial use of the VR system, participants were asked to note whether they experienced any common sickness symptoms associated with VR, such as discomfort, fatigue, vertigo, or dizziness. Responses were provided on a 4-item scale ranging from *none* to *severe*. All measures of feasibility and acceptability were taken during the intervention (eg, attendance) or follow-up (eg, open-response feedback) except for the VRSQ, which was administered to participants before randomization.

#### Social Connection

The relatedness satisfaction and frustration subscales of the Basic Psychological Need Satisfaction and Frustration Scale were used to assess the overall social connection experienced by participants during the 4-week intervention [67,69]. This scale examines the satisfaction and frustration experienced within the relatedness pillar (ie, the extent to which one experiences meaningful social connection) of the SDT postulated by Deci and Ryan [14] and was used as an indicator of social connection. An exemplar satisfaction item is “I feel that the people I care about also care about me,” and an example of a frustration item is “I feel excluded from the group I want to belong to.” Participants were asked to rank their feelings to the statements presented on a scale ranging from 1=*not true at all* to 5=*completely true*. Both subscale scores range from 4 to 25, with higher values indicating greater satisfaction or frustration with their feeling of social connection.

#### Monitoring PA

PA was monitored primarily as a feedback mechanism via the Garmin Vivosmart 4 watch [52]. The data extracted included daily steps, daily minutes of MVPA (ie, time spent at ≥70% of the individual’s maximum heart rate), and total PA. Each metric was calculated for the week before the initiation of the intervention and during the fourth week of the program. We also leveraged weekly minutes of MVPA to determine whether participants achieved their PA prescription (ie, 150 min of MVPA) during the final week of the intervention.

#### Functional Capacity

During the first visit and after a review of past health and current mental status, participants underwent the 6MWT [51]. A previous investigation found the maximum heart rate achieved during the 6MWT to be approximately 80% of a participant’s true maximal heart rate during a graded exercise test on a treadmill [70]. Immediately after the completion of the 6MWT, a study team member assessed heart rate by palpating the participant’s radial pulse for 30 seconds and then multiplying the count by 2. This was used as the participant’s maximal heart rate during the 6MWT. During PA prescription, the maximal heart rate measured during the 6MWT was used to estimate each participant’s maximal heart rate and was used for tailoring each participant’s PA program [70].

### Statistical Analysis

Descriptive statistics, including medians and IQRs for continuous variables and counts and percentages for categorical variables, were computed for baseline participant characteristics as well as feasibility and acceptability measures. Open-ended items were converted from handwritten responses to text files and reviewed for themes and exemplar quotes by KK. In addition, social connection, PA measures, and 6MWT unadjusted baseline and follow-up data are presented by condition numerically and graphically. Finally, to explore the preliminary effect of VR and videoconference delivery on social connection, PA, and functional capacity, we computed Cohen *d* effect sizes (≥0.2=small, ≥0.5=medium, and >0.8=large [71])*.* All analyses were conducted using SPSS software (version 27.0.1; IBM Corp) [72].

### Ethical Considerations

All protocols were reviewed and approved by the Wake Forest University Institutional Review Board (IRB00023881), and all interested and eligible individuals completed an approved informed consent document before study participation. All participants’ data collected during the study were deidentified for data security, and participants were allowed to retain their Garmin Vivosmart 4 as compensation for their time.

## Results

### Participants

Participant characteristics are presented in Table 1. Most of the participants (6/9, 67%) were female, the median age of the participants was 66.0 (IQR 4.8) years, and the median BMI was 32.9 (IQR 8.2) kg/m². Participants tended to have a high degree of education (college or university graduate; 7/9, 78%), and most of the participants identified as White (7/9, 78%), while the remaining participants identified as belonging to >1 race (2/9, 22%).

**Table 1:** Baseline sample characteristics for the virtual reality (VR) and videoconference groups.

Demographics		VR group (n=5)	Videoconference group (n=4)	All participants (n=9)
Sex, n (%)				
	Male	2 (40)	1 (25)	3 (33)
	Female	3 (60)	3 (75)	6 (67)
Age (y), median (IQR)		66.00 (63.60-68.40)	65.50 (63.25-67.75)	66.00 (63.60-68.40)
BMI (kg/m^2^), median (IQR)		40.30 (34.20-46.40)	32.60 (31.95-33.25)	32.90 (28.8-37.00)
Race, n (%)				
	White	5 (100)	2 (50)	7 (77)
	>1	0 (0)	2 (50)	2 (22)
Ethnicity, n (%)				
	Hispanic or Latinx	0 (0)	0 (0)	0 (0)
	Not Hispanic or Latinx	5 (100)	4 (100)	9 (100)
Education, n (%)				
	1-3 years of college or 2-year college, vocational, or technical school	2 (40)	0 (0)	2 (22)
	College or university graduate	0 (0)	2 (50)	2 (22)
	Master’s degree	2 (40)	1 (25)	3 (33)
	PhD or equivalent	1 (20)	1 (25)	2 (22)
Employment status, n (%)				
	Full time	0 (0)	2 (40)	2 (22)
	Retired: working part time	2 (40)	1 (25)	3 (33)
	Retired: not working at all	2 (0)	1 (25)	3 (33)
	Laid off or unemployed	1 (20)	0 (0)	1 (11)
Technology use (d/wk), median (IQR)				
	Smartphone	7.00 (0.00)	7.00 (0.00)	7.00 (0.00)
	Tablet computer	1.00 (3.00)	0.50 (3.00)	1.00 (3.00)
	e-Reader	1.00 (3.00)	0.50 (3.00)	0.00 (3.00)
	Laptop or desktop computer	7.00 (0.00)	6.25 (1.00)	7.00 (0.00)
	Stand-alone video game	0.00 (0.00)	0.00 (0.00)	0.00 (0.00)
Technology self-efficacy (rated on a scale ranging from 1 to 10), median (IQR)				
	Smartphone	0.00 (9.00)	8.30 (3.00)	7.00 (10.00)
	Tablet computer	10.00 (0.00)	9.50 (1.50)	9.50 (2.00)
	e-Reader	10.00 (3.00)	9.50 (1.50)	8.50 (3.00)
	Laptop or desktop computer	5.00 (10.00)	9.00 (2.80)	6.50 (5.00)
	Stand-alone video game	10.00 (0.00)	10.00 (1.30)	10.00 (0.00)

### Feasibility and Acceptability

**Figure 2:**
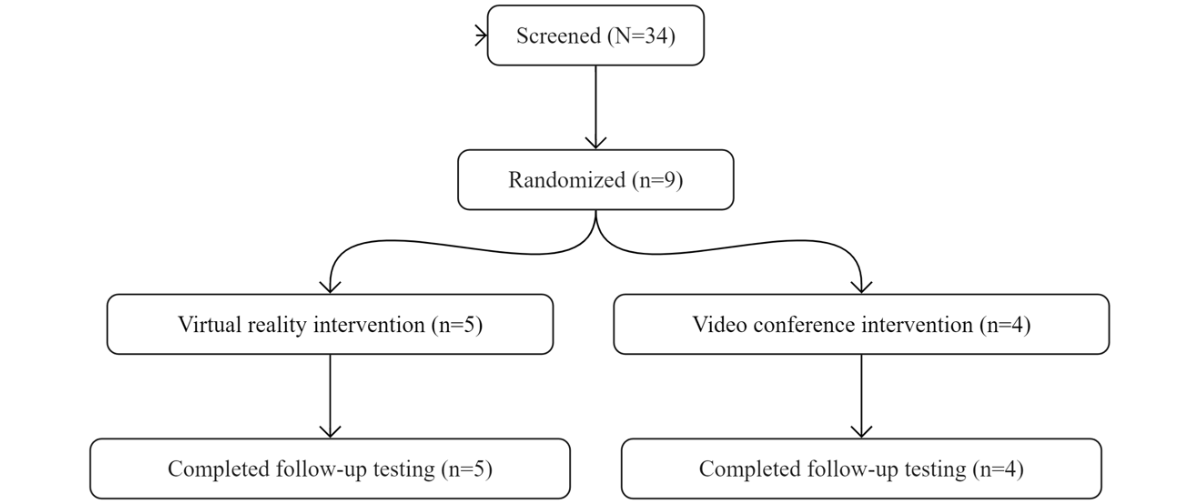
CONSORT (Consolidated Standards of Reporting Trials) flow diagram.

A CONSORT (Consolidated Standards of Reporting Trials) flow diagram depicting recruitment, enrollment, and retention is presented in Figure 2. A total of 34 people were contacted, of whom 9 (26%) were recruited. Overall, 97.2% (105/108) of the coaching meetings were attended (VR group: 59/60, 98%; videoconference group: 46/48, 96%). Of the 5 participants randomized to the VR group, data from only 4 (80%) regarding VR use outside of meeting times were available because the remaining participant misplaced their activity log. The median count of VR sessions engaged in outside of meeting times was 5.5 (IQR 0.5; range 0-27). Notably, 1 (20%) of the 5 VR participants did not use the VR system outside of meetings due to feelings of overstimulation caused by the VR system. There were no adverse events reported in the study.

None of the participants noted *severe* feelings of VR-related sickness, and, thus, none were excluded after the “technology run-in” period. Of the 9 participants who completed the VRSQ, 5 (56%) indicated experiencing any feelings of VR-related sickness. The median VRSQ score of all participants was 2.0 (IQR 3.0) out of a possible 27 points, indicating slight or no VR-related sickness. Furthermore, 44% (4/9) indicated no feelings of sickness or discomfort during or after VR use. Those who were randomized to the VR group and provided activity diary information on their VR use outside of scheduled meetings reported having a median enjoyment rating of 5.0 (IQR 0.0) on a scale ranging from –5 to 5. Group and sample median scores from the Likert responses of the program evaluation are presented in Table 2. While both groups reported similar perceptions of ease when engaging in meaningful dialogue, connecting with their peers on a personal level, and accomplishing goals set by their activity coach, the VR group reported a higher median perception of ease when finding options for PA that they enjoyed (VR group: median 4.0, IQR 1.0; videoconference group: median 2.0, IQR 0.8).

**Table 2:** Results from the program evaluation Likert-style prompts (score range −5 to 5^a^).

Evaluation prompts	Virtual reality group (n=5), median (IQR)	Videoconference group (n=4), median (IQR)	All participants (n=9), median (IQR)
How easy or difficult was it to engage in meaningful dialogue with your peers?^b^	4.0 (0.0)	3.5 (1.0)	4.0 (1.0)
How connected did you feel to your peers on a personal level?^c^	3.0 (1.0)	3.0 (0.5)	3.0 (1.0)
How easy or difficult was it to accomplish goals set by you and your coach?^b^	3.0 (1.0)	3.0 (0.8)	3.0 (1.0)
How easy or difficult do you think it was trying to find options for physical activity that you enjoyed?^b^	4.0 (1.0)	2.0 (0.8)	3.0 (2.0)

^a^Positive values indicate more favorable responses.

^b^Responses range from very difficult to very easy.

^c^Responses range from very disconnected to very connected.

An informal investigation of the open-ended postprogram survey responses suggested that participants found the VR meetings immersive and their VR options to be enjoyable. Regarding the meeting rooms, a participant noted as follows:

Using the [Quest] was so helpful. VR is so much better than Zoom meetings. It felt as though we were actually face-to-face.

VR Participant 4

Another participant also noted that their favorite aspect of the VESPA study was the Quest:

Had never really been into electronics or games. Was lost at first, but started really enjoying, especially Beat Saber.

VR Participant 3

While 4 (80%) of the 5 participants had generally positive reviews with regard to the VR technology used, 1 (20%) offered a contrasting view:

[The Quest had] excessive visual stimulation [and was] overwhelming...I had a near breakdown when first encountering the [Quest] display...Not everyone can accept or enjoy the intensity and immensity of the visual space affected by [the Quest] on the first encounter.

VR Participant 5

Notably, however, this participant attended 100% of the 1-on-1 and group meetings using the VR system.

### Social Connection

**Figure 3:**
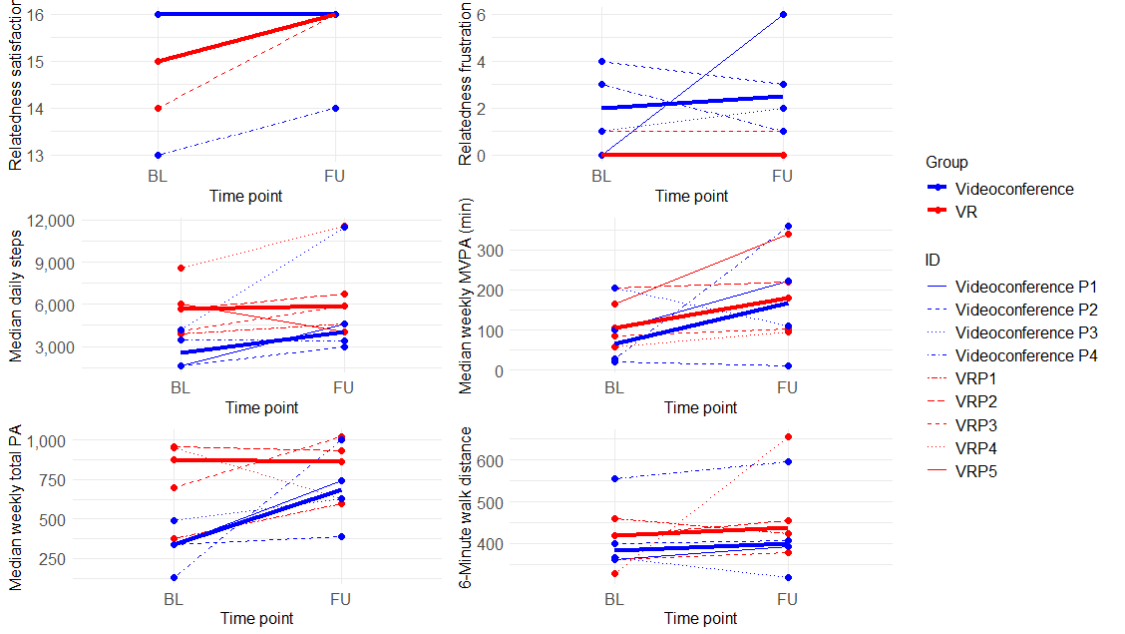
Individual participant median change scores from baseline to follow-up for the relatedness satisfaction and frustration subscales of the Basic Psychological Need Satisfaction and Frustration Scale, median daily steps per day, median minutes of weekly moderate to vigorous physical activity (MVPA), median minutes of total weekly physical activity (PA), and 6-minute walk test distance in meters. A total of 5 participants (virtual reality [VR] group: n=2, 40%; videoconference group: n=3, 60%) reported maximum relatedness satisfaction at both time points and as such are hidden behind the median videoconference group score, while 4 (80%) of the 5 VR group participants reported the minimum score for relatedness frustration and as such are hidden behind the median VR group score. BL: baseline; FU: follow-up; VCP: videoconference group participant; VRP: VR group participant.

Figure 3 shows the changes in individual participant and group scores from baseline to follow-up for the relatedness satisfaction and frustration subscales, and Table 3 shows unadjusted baseline and follow-up median group scores for each group. The results indicated that both groups had consistently high relatedness satisfaction from baseline to follow-up, with, of the 9 participants, 5 (56%; VR group: n=2, 40%; videoconference group: n=3, 60%) reporting the highest relatedness satisfaction at both time points and 4 (44%; VR group: n=3, 75%; videoconference group: n=1, 25%) increasing their scores after the intervention. This resulted in a moderate to large effect of the intervention for the VR group (Cohen *d*=0.73) and a small effect for the videoconference group (Cohen *d*=0.23). Participants in the VR group did not deviate from their original relatedness frustration scores (4/5, 80% reported the lowest score at both time points; Cohen *d*=0.00), while, of the 4 participants in the videoconference group, 2 (50%) indicated experiencing higher relatedness frustration, and 2 (50%) noted experiencing lower relatedness frustration (Cohen *d*=0.67).

**Table 3:** Unadjusted social connection, physical activity (PA), and functional capacity scores at baseline and week 4 follow-up.

Measures		Virtual reality group				Videoconference group		
		Baseline, median (IQR)	Week 4 follow-up, median (IQR)	Cohen *d*	Baseline, median (IQR)		Week 4 follow-up, median (IQR)	Cohen *d*
Relatedness								
	Satisfaction	15.00 (1.00)	16.00 (0.00)	0.73	16.00 (0.80)		16.00 (0.50)	0.23
	Frustration	0.00 (0.00)	0.00 (0.00)	0.00	2.00 (3.00)		2.50 (2.00)	0.67
PA								
	Daily steps	5545.00 (3222.75)	5800.00 (4722.50)	0.35	2589.00 (1968.75)		4092.50 (6766.00)	1.35
	Weekly total (min)	872.11 (415.85)	838.16 (33.74)	0.11	329.24 (252.13)		695.261 (464.97)	1.26
	Weekly MVPA^a^ (min)	108.53 (6.94)	180.91 (178.69)	0.88	64.75 (156.68)		171.68 (288.98)	1.30
Functional capacity								
	6MWD^b^ (m)	420.00 (98.00)	439.00 (93.00)	1.03	382.00 (73.00)		401 (80.00)	0.12

^a^MVPA: moderate to vigorous physical activity.

^b^6MWD: 6-minute walk distance.

### PA Results

Unadjusted group median (IQR) values for daily steps, total weekly PA, and weekly MVPA for baseline and follow-up measures can be found in Table 3 and graphically in Figure 3. The results indicated a small to moderate effect for median daily steps (Cohen *d*=0.35), no effect for median total weekly PA (Cohen *d*=0.00), and a large effect for median weekly MVPA (Cohen *d*=0.88) for the VR group. Conversely, the results indicated large effects for the videoconference group for median daily steps (Cohen *d*=1.35), median total weekly PA (Cohen *d*=1.26), and median weekly MVPA (Cohen *d=*1.30). Notably, while both groups had similar rates of participants meeting the PA prescription during week 4 (VR group: 3/5, 60%; videoconference group: 2/4, 50%), the videoconference group’s median total weekly PA doubled and median weekly MVPA nearly tripled.

### Functional Capacity

The group median distance traveled for the 6MWT at baseline and follow-up can be found in Table 3 and graphically in Figure 2. The results indicated that the VR group experienced a large effect for the distance walked during the 6MWT, whereas the videoconference group experienced no effect after the intervention (VR group: Cohen *d*=1.03; videoconference group: Cohen *d*=0.12). Furthermore, median distances increased by 19 meters for both groups after the intervention, which is indicative of a small but clinically meaningful change [49].

## Discussion

### Principal Findings

The VESPA study found that VR is an acceptable medium for delivering a theory-based group-mediated PA intervention and may offer unique benefits compared to videoconference. This is crucial because a previous systematic review on VR interventions for older adults’ health outcomes found that only 1 study reported on the usability and acceptability of VR-delivered interventions [46]. Given that videoconference has already been shown to be feasible and acceptable for PA interventions in older adults, the potential of VR as an alternative or complementary tool for promoting PA is promising [73-75]. Importantly, all older adult participants found the Quest headset acceptable, as indicated by low feelings of VR-related sickness. This is encouraging because there has been concern about VR-induced nausea, which is common in older VR devices [40,50,68,76]. We also found that remote delivery of a group behavioral intervention via VR was feasible, as indicated by similarly high attendance rates in both the VR and videoconference groups, with no adverse events reported in either condition. However, heterogeneity in the uptake of VR was noted. The research team observed that some of the participants (2/5, 40%) used the VR headset every 2 days, although 1 (20%) of the 5 VR group participants only used it for meetings with the coach and group. Notably, the postintervention evaluations showed that the VR group participants consistently had ease when searching for enjoyable PA (both in-person and VR PA). While it is unclear whether this ease was solely due to VR, it is encouraging that most of the participants (4/5, 80%) used the system for PA during the intervention. Open-ended program evaluations revealed that participants felt more immersed and connected with other users during the intervention, while 1 (20%) of the 5 VR group participants noted experiencing overstimulation when first using the Quest headset. Strategies to improve participant uptake of the VR system are discussed herein.

In addition to investigating the feasibility and acceptability of VR for remote PA intervention among older adults, we also investigated how VR delivery affected the key social outcomes within SDT (ie, relatedness satisfaction and frustration), PA, and functional capacity. We would like to emphasize that these are exploratory findings from a small sample, unsuitable for hypothesis testing. However, these descriptive results are valuable for informing future research with larger, more diverse samples. With this limitation in mind, we anticipated amplified effects on social outcomes, given the role of presence in VR design and its impact on fostering social connection. The results indicated that both groups maintained high levels of relatedness satisfaction (ie, feelings of connectivity and group membership), while only the VR group maintained low levels of relatedness frustration (ie, feelings of exclusion or isolation; Figure 3). This is exciting because many of the features offered by contemporary VR headsets were not used (eg, social “meetups” and multiplayer active games). It can be speculated that additional presence-inducing features could enhance the social experience for the VR group. However, a participant found the immersion unsettling, suggesting that some may benefit more from low- or nonimmersive environments because similar relatedness satisfaction and frustration levels were observed in both the VR and videoconference groups.

While the weekly MVPA results indicated large effects for both groups (VR group: Cohen *d*=0.88; videoconference group: Cohen *d*=1.30), all PA-related outcomes favored the videoconference group. This discrepancy is likely due to a multitude of factors, such as the videoconference group having approximately 45 fewer minutes of baseline weekly MVPA, some VR group participants failing to progress beyond early game difficulty levels, and VR group participants engaging in short-term PA sessions using the VR system that did not get identified as PA bouts. However, a large increase in PA behaviors in the videoconference group was expected because previous investigations have found videoconference to be an effective medium for delivering group-mediated PA interventions to older adults [73-75]. Therefore, the PA-related effects from the VR group are encouraging and indicate a need for further exploration of how VR can be used to facilitate PA behaviors in older adults. Strategies for encouraging participants to increase active game intensity and use longer active game options are provided herein.

Both groups traveled a median distance of 19 meters further than baseline, which indicates a small but clinically meaningful change [77]. However, the results indicated a large effect (Cohen *d*=1.03) for the VR group and no effect (Cohen *d*=0.12) for the videoconference group. The difference in effects seen in the 6-minute walk distance test could be due to the small sample size, considerable differences in step behaviors at both time points (Table 3), or the incorporation of coordination training in active games, which may have had a unique impact on the functional capacity of older adults [78-80]. In the future, more adequately powered studies should investigate this difference and explore whether the time spent engaging with VR active games of differing intensities correlates with change in the 6-minute walk distance test scores.

### Key VR-Based Future Modifications

#### Overview

It is promising that VR was feasible and acceptable and associated with positive initial results in the domain of social connection. As expected, several limitations to the protocol emerged in this first trial. To guide other teams interested in deploying VR-mediated activity interventions, we present an inventory of key limitations and proposed modifications here. Notably, these lessons are not exclusive to VR. While VR has proven effective for enhancing presence, future investigations of other technologies, such as XR, which also aim to enhance presence, may benefit from the lessons outlined in the following subsections.

#### Use of Social Features

Participants in the VR group did not fully use the social features available, such as adding friends or meeting in virtual rooms. It can be speculated that meeting outside of scheduled times for social or PA-related purposes could enhance their sense of connectedness. As this pilot focused on comparing VR and videoconference for intervention delivery with a coach, participants were only encouraged to use VR for scheduled meetings and active games. However, VR systems now offer a growing range of social tools, including friends’ lists and active social gaming. Future trials should assess whether these features further enhance the perceived connection between older adult users.

#### Active Game Modifications

During 1-on-1 meetings, the behavioral coach noted that many participants did not adjust the active game settings to match their improving abilities or only used 1 active game mode within a specific active game (eg, story mode vs exercise mode within *Creed: Rise to Glory*) [57]. While directions were provided to the participants regarding adapting the active games to their physical abilities and skill levels, many did not deviate from the initial low-intensity settings. In light of this, behavioral coaches delivering similar interventions in the future should frequently inquire about gameplay settings and how they relate to the participant’s current comfort with the active game, progression within the game intensity options, and their overall volume of MVPA.

#### Tailoring Experience

As a participant noted, not all VR users can handle the intensity of VR immersion, and some may feel overwhelmed. To reduce the risk of overstimulation for VR-naïve participants, future studies should regularly assess participants’ comfort before and during the run-in session and adjust accordingly. If discomfort or anxiety is reported, study teams should offer incremental immersion options (eg, no volume initially and starting in a seated position) to help ease participants into VR. It is also important that methods to meet participants’ needs are continually updated as technology evolves; for instance, as XR systems emerge on the market, passthrough interfaces that anchor interface elements to a person’s real environment may help to mitigate overstimulation. In addition, while it is possible that some VR features or applications not used in this study may not be well suited for older individuals due to low familiarity or cognitive challenges, researchers may leverage a third party (eg, a participant’s adult child or an activity coach) to assist older adults in overcoming any challenges and facilitate proper use. By doing so, older adults will be empowered to use this technology and adequately prepared to adapt to the evolving VR landscape.

### Strengths and Limitations

The predominant strength of this study is that it provides guidance for the delivery of future theory-driven, social, and remote PA interventions using contemporary and quickly evolving technology. While the results indicate that participants were amenable to the use of VR technology, the feedback from the participants and behavioral coach provides useful insight into how similar interventions in the future can be adapted to better leverage the technology. Another strength of this investigation is the use of commercially available technology. To date, health interventions using VR technologies have primarily targeted issues such as pain, anxiety, and posttraumatic stress disorder and are now being used to improve health outcomes in older adults [46,76,81-84]. These lines of research usually require modifications to the user’s real-world environment, VR system, or the use of applications designed specifically for the study that are rarely accessible after completion of the research period. Furthermore, there has been a lack of research using commercially available VR technology for at-home social or active purposes. Our use of such technology allows future researchers to build directly on our lessons learned. However, there are important limitations to address. The goal of this trial was a rapid initial evaluation of the feasibility and acceptability of VR-delivered activity programming for older adults, and the sample size was not large enough for hypothesis testing. A key next step is to develop an intervention that is adequately powered to detect group differences in the social, PA, and physical function outcomes of interest. Another limitation of this study is that the intervention lasted only 4 weeks. A review of 8 PA programs for balance and fall prevention in older adults found that the shortest intervention duration was 8 weeks [84]. We suspect that a longer intervention duration would allow the participants to become more familiarized with the technology and, in turn, use the technology more often for social and active purposes. Future work should investigate longer durations of interventions and no-contact follow-up periods to study the effects of similar interventions.

### Conclusions

The results of this small pilot trial support that contemporary VR systems (1) are feasible to use in an older adult population, (2) are acceptable to older adults for meeting and activity purposes, (3) do not support previous notions regarding VR-related sickness, (4) provide socially rich meeting platforms for its users, and (5) offer older adults legitimate methods of engaging in MVPA. This is an exciting avenue of research because the use of VR in the general population and in research is in its infancy. Recognizing that VR technology—and analogous technologies such as XR—is evolving rapidly, the time is right to adopt a careful, iterative, and rapid approach to developing VR-delivered health behavior interventions.

## Appendix

Multimedia Appendix 1 CONSORT-EHEALTH (Consolidated Standards of Reporting Trials of Electronic and Mobile Health Applications and Online Telehealth) checklist (V 1.6.1).

## Abbreviations

6MWT: 6-minute walk test
CONSORT-EHEALTH: Consolidated Standards of Reporting Trials of Electronic and Mobile Health Applications and Online Telehealth
CONSORT: Consolidated Standards of Reporting Trials
MVPA: moderate to vigorous physical activity
PA: physical activity
SDT: self-determination theory
VESPA: Virtually Engaging Socially with Physical Activity
VR: virtual reality
VRSQ: Virtual Reality Sickness Questionnaire
XR: extended reality
